# Time to Surgery for Distal Radius Fractures: A Quality Improvement Audit

**DOI:** 10.7759/cureus.96566

**Published:** 2025-11-11

**Authors:** Nicholas A Clausen, Munzir Akasha, Yousif Mohamed, Petr Jemelik, David O'Briain

**Affiliations:** 1 Department of Trauma and Orthopedic Surgery, University Hospital Waterford, Waterford, IRL

**Keywords:** clinical audit, distal radius fractures, evidence-based practice, orthopedic surgery, quality improvement and patient safety

## Abstract

Background: The British Orthopedic Association Standards for Trauma (BOAST) recommend early surgery for distal radius fractures, giving priority to intra-articular fractures over extra-articular ones. Delays increase surgical complexity, risk of malunion, and poorer outcomes. Timely surgery improves results, resource use, and care pathways.

Patients and methods: A retrospective analysis of patients undergoing surgical management of distal radius fractures was conducted over three months (from July to September 2023; n = 53 patients) at a regional hospital and compared with BOAST guidelines. Demographics, injury, and intervention data were recorded. Following staff education, a second cycle was undertaken (from April to June 2024; n = 57 patients). A third cycle (from October to December 2024; n = 51 patients) assessed the impact of our interventions on sustaining improvement. Time to operative management was measured by date of injury (DOI) and date of referral (DOR). Proportions of extra- or intra-articular fractures operated on within timelines were statistically assessed with either chi-square or Fisher's test, and trends were analyzed using Cochran-Armitage trend analyses.

Results: In total, 161 operated distal radius fractures were analyzed. Following the introduction of staff education and changes to departmental trauma lists, there were nonsignificant improvements in intra-articular fractures operated on within three DOI across the three cycles (first: 59%, second: 56%, third: 75%; p = 0.2665; p(trend) = 0.1924). Extra-articular fractures operated within seven DOI worsened across the three cycles (first: 92%, second: 97%, third: 68%; p = 0.0119; p(trend) = 0.0243). Intra-articular fractures operated within three DOR had slight fluctuations (first: 74%, second: 68%, third: 88%; p = 0.1923; p(trend) = 0.1975). Extra-articular fractures operated within seven DOR remained stable (first: 92%, second: 100%, third: 89%; p = 0.1596; p(trend) = 0.8057).

Conclusion: Treatment of intra-articular distal radius fractures within three DOI or three DOR did not show statistically significant improvements following staff education and service changes. Improved identification and prioritization of intra-articular injuries was observed. In contrast, the proportion of extra-articular fractures operated on within seven DOI differed significantly between audit cycles and exhibited a significant downward trend. No other outcome showed a statistically significant difference or trend. The reduction in extra-articular fractures operated within seven DOI (68%) compared to seven DOR (89%) in the third audit cycle may be explained by patients presenting late or delays in transfers or referrals from peripheral hospitals. Implementation of routine education sessions regarding BOAST guidelines at staff turnover may contribute to improved operative rates within both cohorts. Further audit cycles are necessary to assess the continued impact of service changes on these operative timelines.

## Introduction

Distal radius fractures are some of the most commonly encountered fractures [1]. These fractures can be subdivided into several categories, but two broad categories involve extra- or intra-articular fractures. Regardless of the technique used to treat a distal radius fracture, appropriate reduction and restoration of native anatomy are necessary to achieve outcomes with the goal of a return to mobility, function, work, and/or sport within reasonable timeframes [1]. Intra-articular fractures damage articular cartilage and frequently lead to osteoarthritis, articular pain, decreased joint function, and disability [2]. Current treatment for intra-articular fractures focuses on restoring joint surface congruity as articular surface contact stresses and/or instability increase with time [3].

Distal radius fractures require timely surgical intervention, as outlined by the British Orthopedic Association guidelines, which recommend surgery within 72 hours for intra-articular fractures and within seven days for extra-articular fractures [4]. Delayed surgery increases the likelihood of malunion, joint incongruity, and surgical complexity, prolonging rehabilitation and yielding suboptimal results [3]. Delays in operative management within these timelines may happen for a variety of reasons: the fracture is viewed as a relatively minor injury in a patient who can cope at home while awaiting surgery, complex fractures occasionally require CT imaging (likely those in the intra-articular cohort), waiting for an upper limb specialist to become available, or lack of awareness whether or not a delay may significantly affect a patient’s long-term outcome [5].

Patients with intra-articular distal radius fractures with longer time to surgery are more likely to experience an early postoperative complication [6]. Early surgery is associated with improved short- and long-term Disabilities of the Arm, Shoulder and Hand scores [7]. Surgery within seven days of the fracture event has been shown to have lower surgeon-perceived difficulty and led to better surgeon-perceived reduction quality following fixation [8]. Adherence to British Orthopedic Association Standards for Trauma (BOAST) guidelines optimizes resource utilization and streamlines care pathways. This audit aims to evaluate current distal radius management practice within our department, identify areas for improvement, and implement strategies to enhance adherence to recommended timeframes, ultimately improving patient care efficiency and outcomes.

In a busy regional trauma center, distal radius fractures scheduled for surgery are treated as day cases. Organization and prioritization are foreseen by trauma coordinators without adherence to any defined criteria. The daily orthopedic trauma list is managed using an Excel spreadsheet (Microsoft Corporation, Redmond, WA) that includes a dropdown entry for “distal radius fracture” without specifying articular involvement. This lack of distinction led to inconsistent prioritization, with some intra-articular fractures not operated on within the recommended 72-hour window, contrary to BOAST guidelines.

The department recognized the need for improved standardization and clearer triage. This quality improvement (QI) project aimed to improve timely operative management of intra-articular distal radius fractures by introducing targeted staff education and modifying the trauma list to highlight articular involvement.

## Materials and methods

This was a retrospective study of 161 operated distal radius fractures analyzed across three audit cycles. This QI study was approved by the University Hospital Waterford Quality and Patient Safety Department (Registration number A2410). Patients aged 18 years or older presenting to our department with a distal radius fracture who underwent operative management were included in the study. Patients with multiple fractures, treated conservatively, requiring additional imaging (e.g., CT or MRI), or children were excluded. Articular classification was determined by radiographic review by a consultant radiologist.

In the initial audit cycle, a total of 53 operated distal radius fractures over three months (from July to September 2023) were compared with the BOAST guidelines for the management of distal radius fractures [4]. Microsoft Excel was used for data collection, including fracture type (intra-articular vs. extra-articular), date of injury (DOI), date of referral (DOR), and number of days awaiting surgery. The aim was to ensure timely management of intra- and extra-articular fractures within desired timelines to avoid complications. In our department, we agreed on an acceptable margin of 80% or more for both extra-articular and intra-articular fractures, as neither the BOAST guidelines nor studies in our literature search commented on this. In the initial cycle, extra-articular fractures operated within one week were excellent (>80%), while intra-articular fractures operated within 72 hours were unsatisfactory (<80%). Additionally, extra-articular fractures operated on within 72 hours were noted. We planned to continue using the same Excel sheet as a data-collection tool to measure operative management rates in upcoming cycles. We used the Standards for QUality Improvement Reporting Excellence (SQUIRE) reporting guideline [9] to draft this manuscript and the SQUIRE reporting checklist [10] when editing.

Upon completion of the initial audit cycle, a QI team, consisting of a consultant, registrar, and intern, was established. Staff education on the BOAST guidelines, aimed at both doctors and trauma coordinators, was subsequently implemented following the results of the initial audit cycle. Staff attendance was tracked through sign-in sheets, and audit results were presented upon completion of each audit cycle. A second audit cycle of 57 operated distal radius fractures took place over three months (from April to June 2024). After completion of the second cycle, to further shift attention to managing intra-articular fractures within the desired timeline, repeat education and changes to the departmental trauma list were enacted to improve clarity. To facilitate this, the trauma Excel spreadsheet was modified by updating the dropdown list to subcategorize distal radius fractures into extra-articular and intra-articular fractures (Table 1), making it easier for prioritization by the trauma coordinators. A third audit cycle of 51 operated distal radius fractures took place over three months (from October to December 2024). Statistical analysis was performed using Prism (GraphPad Prism, version 10.5.0 for Mac, GraphPad Software, Boston, Massachusetts; www.graphpad.com) using chi-square or Fisher's exact tests and Cochran-Armitage trend tests to compare proportions.

**Table 1:** Comparison of Excel spreadsheet options and changes to the departmental trauma list during the quality improvement project

Time	Before October 2024	After October 2024
Trauma list options	23: radius: distal segment	23A: radius: distal segment intra-articular
	23: ulna: distal segment	23B: radius: distal segment extra-articular
		23: ulna: distal segment

**Figure 1:**
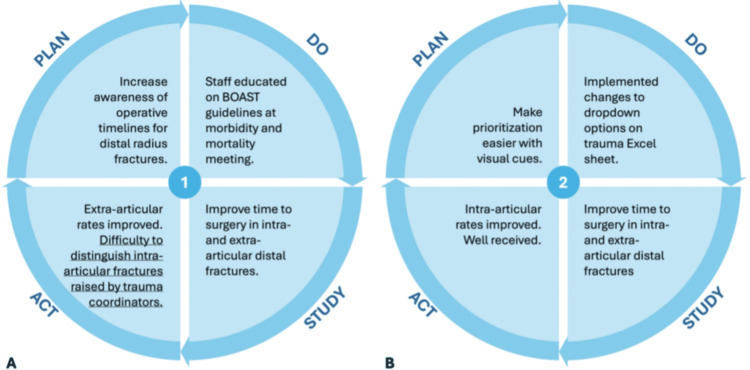
PDSA cycles during the quality improvement project. (A) PDSA Cycle 1 after completion of the first audit cycle. (B) PDSA Cycle 2 after completion of the second audit cycle PDSA: Plan-Do-Study-Act; BOAST: British Orthopedic Association Standard for Trauma

Two plan-do-study-act (PDSA) cycles were completed over nine months following completion of the first and second audit cycles (Figure 1). PDSA cycle 1 (Figure 1A) occurred before the initiation of the second audit cycle. For the initial intervention, staff education was implemented at the morbidity and mortality meeting. The BOAST guidelines were discussed with all attending consultants, doctors, and trauma coordinators. After analysis of operative rates within the second cycle and careful discussion with the trauma coordinators, it was agreed to implement changes to the trauma list, and PDSA cycle 2 (Figure 1B) was completed before initiation of the third audit cycle. The dropdown list in the trauma Excel spreadsheet was edited to include two separate options for distal radius fractures to further elucidate articular involvement.

## Results

A total of 161 operated distal radius fractures were analyzed across the three audit cycles, including 77 (48%) extra-articular fractures and 84 (52%) intra-articular fractures (Table 2). In the first cycle, 92% (24/26) of extra-articular fractures were operated on within one week of injury and 42% (11/26) within 72 hours of injury, while 59% (16/27) of intra-articular fractures were operated on within 72 hours of injury. During the second cycle, 97% (31/32) of extra-articular fractures were operated on within one week of injury, and 63% (20/32) were operated on within 72 hours of injury. Rates of intra-articular fractures operated on within 72 hours of injury were similar to the first cycle at 56% (14/25). In the third cycle, the only cohort with improvements was intra-articular fractures operated on within 72 hours of injury at 75% (24/32). There was a notable reduction in extra-articular fracture caseload and rates of extra-articular fractures operated on both within seven days of injury and 72 hours of injury deteriorated (68% and 32%, respectively) compared to the previous two cycles. Assessing the trend across all three audit cycles, there were nonsignificant improvements in intra-articular fractures operated within three DOI (first: 59%, second: 56%, third: 75%; p = 0.2665; p(trend) = 0.1924). Extra-articular fractures operated within seven DOI worsened across the three cycles (first: 92%, second: 97%, third: 68%; p = 0.0119; p(trend) = 0.0243). Extra-articular fractures operated within three DOI varied across the three cycles (first: 42%, second: 63%, third: 32%; p = 0.0787; p(trend) = 0.6229).

**Table 2:** Summary of operative rates for extra-articular and intra-articular distal radius fractures from the first, second, and third audit cycles by date of injury and date of referral DOI: days of injury; DOR: days of referral

Distal radius fractures	First cycle, n (%)	Second cycle, n (%)	Third cycle, n (%)	X^2^/Fisher's p value	p(trend)
Total number of cases	53	57	51	-	-
Number of extra-articular fractures	26/53 (49%)	32/57 (56%)	19/51 (37%)	-	-
Number of intra-articular fractures	27/53 (51%)	25/57 (44%)	32/51 (63%)	-	-
Extra-articular operated within seven DOI	24/26 (92%)	31/32 (97%)	13/19 (68%)	0.0119	0.0243
Extra-articular operated within three DOI	11/26 (42%)	20/32 (63%)	6/19 (32%)	0.0787	0.6229
Intra-articular operated within three DOI	16/27 (59%)	14/25 (56%)	24/32 (75%)	0.2665	0.1924
Extra-articular operated within seven DOR	24/26 (92%)	32/32 (100%)	17/19 (89%)	0.1596	0.8057
Extra-articular operated within three DOR	12/26 (46%)	23/32 (72%)	11/19 (58%)	0.1366	0.3300
Intra-articular operated within three DOR	20/27 (74%)	17/25 (68%)	28/32 (88%)	0.1923	0.1975

Operative rates of extra- and intra-articular distal radius fractures between DOR and date of surgery were also analyzed. Extra-articular fractures operated within seven DOR remained stable (first: 92%, second: 100%, third: 89%; p = 0.1596; p(trend) = 0.8057). Intra-articular fractures operated within three DOR had slight fluctuations (first: 74%, second: 68%, third: 88%; p = 0.1923; p(trend) = 0.1975). Extra-articular fracture operative rates within three days of referral varied between the first, second, and third cycles (46%, 72%, and 58%, respectively). Extra-articular fractures operated within three DOR varied across the three cycles (first: 46%, second: 72%, third: 58%; p = 0.1366; p(trend) = 0.3300).

## Discussion

This QI project sought to enhance adherence to BOAST guidelines for operative management of distal radius fractures, with particular focus on intra-articular fractures. The PDSA cycles within each audit cycle were associated with a nonsignificant improvement in the rate of intra-articular fractures operated within 72 hours of injury, from 59% in the first cycle to 75% in the third cycle. Although this was below our acceptable margin of 80%, we noted in our analysis of intra-articular fractures operated within 72 hours of referral that rates increased from 74% in the first cycle to 88% in the third cycle.

The most impactful intervention was the modification of the trauma list to visually distinguish intra-articular from extra-articular fractures. This low-cost, easily implemented change addressed a key barrier identified in earlier cycles: difficulty for trauma coordinators in recognizing and prioritizing articular involvement. It is possible that the lack of a visual cue following PDSA cycle 1 may have resulted in the increase of extra-articular fractures operated within 72 hours of injury in the second cycle (63%) compared to the first cycle (42%), while intra-articular fractures operated within 72 hours of injury were similar between the two cycles. By providing a simple visual cue within the existing scheduling system, this intervention streamlined case prioritization and directed operative resources to the highest priority cases. Recurring staff education sessions were also important in reinforcing the rationale for the BOAST timelines. Although improvements following PDSA cycle 1 were modest, the combination of repeated education with a systemic workflow change in the PDSA cycle 2 likely had a synergistic effect, reflected in the substantial improvement in intra-articular operative rates by the third cycle. An unanticipated finding was the decline in extra-articular fractures operated on within both 72 hours and seven days of injury in the third cycle. Several factors may explain this trend, including potential reallocation of theater time to higher priority intra-articular cases or delays in presentation/referral from peripheral hospitals.

A key strength of this project was the pragmatic, low-cost nature of the interventions, which were implemented without disrupting existing workflows. However, several limitations should be acknowledged. Staff education is inherently transient in impact due to the frequency of nonconsultant doctor changeover occurring twice a year in our institution, which necessitates regular reinforcement to sustain improvement. Sample sizes were relatively small and varied across the audit cycles, most notably with extra-articular fractures in the third cycle. The project was also limited by factors beyond departmental control, such as delays in referrals or transfers from peripheral hospitals, which can prolong the time to surgery despite internal improvements. Furthermore, this was a single-center study, and results may not be directly generalizable to institutions with different trauma list management systems. Future work should focus on integrating guideline education into staff induction and reauditing to assess the sustainability of improvements.

## Conclusions

The study demonstrates a close adherence to BOAST guidelines in the management of distal radius fractures. It is noted that intra-articular fractures operated on within three DOI and three DOR increased, suggesting an improvement in the identification and prioritization of intra-articular fractures. However, this improvement across the three audit cycles was not statistically significant. In contrast, the proportion of extra-articular fractures operated on within seven DOI differed significantly between audit cycles and exhibited a significant downward trend. The reduction in extra-articular fractures operated within seven DOI (68%) compared to seven DOR (89%) in the third audit cycle may be explained by patients presenting late or delays in transfers or referrals from peripheral hospitals. Implementing routine education sessions on BOAST guidelines at staff turnover may improve operative rates in both cohorts. Further audit cycles are necessary to assess the continued impact of service changes on these operative timelines and the significance of their trends.
